# Effect of the ACTN-3 gene polymorphism on functional fitness and executive function of elderly

**DOI:** 10.3389/fnagi.2022.943934

**Published:** 2022-09-09

**Authors:** Jessica Salles Henrique, Pedro Luiz Garcia Braga, Sandro Soares de Almeida, Nathalia Santanna Petraconi Nunes, Izabelle Dias Benfato, Ricardo Mario Arida, Camila Aparecida Machado de Oliveira, Sérgio Gomes da Silva

**Affiliations:** ^1^Department of Physiology, Universidade Federal de São Paulo, São Paulo, Brazil; ^2^Department of Psychogerontology, Instituto Educatie de Ensino e Pesquisa, Mogi das Cruzes, Brazil; ^3^Hospital Israelita Albert Einstein, São Paulo, Brazil; ^4^Department of Physical and Functional Performance, Universidade Ibirapuera, São Paulo, Brazil; ^5^Department of Bioscience, Universidade Federal de São Paulo, Campus Baixada Santista, Santos, Brazil; ^6^Department of Research and Extension, Fundação Cristiano Varella, Muriaé, Brazil; ^7^Centro Universitário FAMINAS, Muriaé, Brazil

**Keywords:** aging, ACTN-3, functional fitness, executive function, cognition

## Abstract

During aging, physical integrity and cognitive abilities, especially executive function, become compromised, directly influencing the quality of life of the elderly. One good strategy to ensure healthy aging is the practice of physical exercise. Activities to improve aerobic capacity and muscle strength are extremely important in old age. However, some genetic factors can interfere both positively and negatively with these gains. In this context, the polymorphism rs1815739 (R577X) of the α-actinin 3 gene (ACTN-3) is commonly studied and related to muscle phenotype. Thus, the present study aimed to investigate the effect of the ACTN-3 gene polymorphism on the functional fitness (measured by the Senior Fit test) and cognitive capacity (evaluated by the Stroop test) of the elderly (*n* = 347), both men and women. We did not find the effect of genotype on functional fitness, but we did observed a positive effect of the ACTN-3 gene polymorphism on executive function. The presence of the X allele of the ACTN3 gene in the elderly was related to a better performance in the Stroop test (shorter answer time). Our results showed that ACTN-3 gene polymorphism affects the executive function of the elderly but not their functional fitness.

## Introduction

Population aging is increasing worldwide. It is estimated that by 2050, the prevalence of individuals over 60 years of age will be twice as high as it is today. As a result, elderly individuals will represent about 2 billion of the world population (He et al., 2016). However, longevity is not always associated with a good quality of life. In fact, several comorbidities emerge from the aging process that negatively impact aged individuals, as their functional abilities degrade (Blazer et al., 2015). Central nervous system damage, for example, accompanied by cognitive deficits is very common in the aged population (Erickson et al., 2019). Even individuals who are not affected by pathological conditions (e.g., dementias) experience cognitive decline as a result of aging (Harada et al., 2013).

Cognitive decline begins with small cellular damages that in the long term extend to structural changes, such as gray matter atrophy and loss of synaptic density between brain areas (Oliveira et al., 2018; Erickson et al., 2019). In the prefrontal cortex, for example, it is possible to observe the loss of cellular bodies in early adulthood (Fjell et al., 2017). Damages to the prefrontal cortex result in cognitive deficits mainly related to executive functions (EF) (Zanto and Gazzaley, 2019). EF refer to the abilities to successfully perform independent, appropriate behavior, with a purpose of interest (Diamond, 2013). This includes a wide range of cognitive skills such as inhibitory control, selective attention, working memory, and cognitive flexibility (Diamond, 2013).

Changes in EF can be assessed in different ways. The Stroop test, for example, is a widely used tool to measure the central aspects of EF and to predict age-related cognitive decline (Fjell et al., 2017; Scarpina and Tagini, 2017). This test easily assesses the ability to inhibit interference, selective attention, and cognitive flexibility, related to frontal lobe dysfunctions that occur when processing a response to one stimulus simultaneously affects the processing of another response to the same stimulus (Fjell et al., 2017; Scarpina and Tagini, 2017).

Although cognitive functions become compromised during aging, there is robust evidence that the brain is capable of maintaining or recovering some functionality. One of the most commonly used tools to ensure cognitive integrity is physical activity (Blondell et al., 2014; Schlosser Covell et al., 2015; Veronese et al., 2016). Physical activity practice is a low-cost intervention, very effective in controlling age-related complications (Rebelo-Marques et al., 2018). The preservation of cognitive function from physical activity occurs through several molecular and cellular changes (e.g., increased levels of neurotrophic factors, decreased neuroinflammation, induction of neurogenesis, etc.), which influence neural systems involved in cellular signaling, information processing, memory retention, and learning (Loprinzi and Kane, 2015; Gomes da Silva et al., 2016; Henrique et al., 2018). It is important to emphasize that the protective effect of physical activity on the human brain occurs even when the practice starts after the brain maturation phase (Ngandu et al., 2015; Prakash et al., 2015; Tolppanen et al., 2015). Indicating that the brain remains plastic enough to receive the benefits of an active lifestyle.

Besides cognitive protection, engaging in physical practice also ensures a better physical condition for aged individuals. During the aging process, the variables of functional fitness (strength, endurance, agility, and flexibility) tend to decrease naturally, compromising the independence of the individual in carrying out daily tasks (Legrand et al., 2014; Rebelo-Marques et al., 2018). For individuals aged 60 years or more it is highly recommended the practice muscle strengthening activities. This is because good muscle condition prevents sarcopenia and ensures better functional fitness—an individual's ability to perform activities of daily living (ADLs) safely and independently without the early onset of fatigue (Miljkovic et al., 2015; Erickson et al., 2019). Interestingly, muscle strength is also associated with cognitive response in the elderly. For instance, high levels of isokinetic strength are related to better performance of general cognitive function assessed by the Mini-Mental State Examination (MMSE) (Nakamoto et al., 2012). Upper and lower limb strength is also associated with higher scores on cognitive test batteries (Steves et al., 2016), while low levels of muscle mass and strength are associated with poor cognitive performance (Herold et al., 2019).

Genetic factors influence muscle characteristics. For instance, the ACTN-3 R577X polymorphism is commonly related to muscle phenotype. The ACTN3 gene is responsible for encoding the α-actinin-3 protein (Zempo et al., 2010; Pickering and Kiely, 2018). Actinin-3 is a structural protein, a component of the Z disk of muscle fiber, which acts to anchor actin filaments within the sarcomere (Yang et al., 2011). Its expression is restricted to skeletal muscle (type II, fast glycolytic fibers) and, as a consequence, individuals with a deficiency in this protein tend to have a lower percentage of these fibers (Vincent et al., 2007). The R577X SNP results in a C to T base substitution, which leads to the alteration of an arginine base (R) to a premature stop codon (X) (North and Beggs, 1996; North et al., 1999; Yang et al., 2003; Pickering and Kiely, 2018). An estimated 20–30% of the world population (over 1 billion people worldwide), are deficient in α-actinin-3 (North et al., 1999; Mills et al., 2001; MacArthur et al., 2007). There are three possible genotypes: RR and RX—which seem to confer muscle strength predisposition to the individual and XX—which is the total absence of the protein and it is more related to decreased strength and loss of muscle function (Mills et al., 2001; Clarkson et al., 2005; Pickering and Kiely, 2018). These muscle phenotype differences are observed in elite athletes, where individuals who are homozygous for the X allele are underrepresented in speed, power, and strength modalities (Papadimitriou et al., 2019).

Taking into account the important role of muscle strength in healthy aging, we aimed to investigate the effect of the R577X polymorphism of the ACTN-3 gene on functional fitness and executive function in aged individuals. Functional fitness was assessed using the Senior fit test protocol. For the executive function response, the Stroop test was used. Other variables such as years of educational level, anthropometric measurements, and health information were also collected for analysis. Understanding the factors that compromise executive functions in old age can help us to create strategies that can be adopted to increase the functional independence and quality of life of the elderly.

## Materials and methods

We conducted a cross-sectional study of convenience and non-probabilistic sampling approved by the ethics committee of the Federal University of São Paulo (CEP/UNIFESP – CAAE: 0630/2018). To be included in this study, the person had to sign an Informed Consent Form and a medical authorization (issued for up to 1 year) to perform physical tests (requirement of elderly centers). As for exclusion criteria, those who had musculoskeletal and/or neurological diseases that made it impossible to perform the functional capacity tests or were being treated for cognitive, psychological, or psychiatric illnesses and alcoholism were excluded. Therefore, 347 individuals, aged 60 years or more, of both sexes were recruited from an elderly center in the Alto Tiete region of São Paulo state.

### Questionaries and anthropometric data

It was asked through questionaries, information regarding educational level, 12-month fall recall, and general health was provided. Body weight and height were collected to determine body mass index (BMI) by the following equation:


(1) $$\begin{array}{r} \text{BMI}=\frac{\text{Weight}}{\text{Heigh}{\text{t}}^{2}} \end{array}$$

### General functional fitness index

The functional fitness of the volunteers was evaluated by the Senior Fit test method, which is composed of a battery of tests that includes measures of strength, aerobic resistance, flexibility, agility, and dynamic balance. The score is established according to the criteria of each test. Each test has a rating of five categories, according to the participant's performance, gender, and age. In the end, GFFI—an indicator calculated through the sum of the points obtained in each test—is generated. The general classification is ranked from worst to best, receiving the following classifications: very weak, weak, regular, good, and very good.

For our analyses, the categories very weak and weak, and good and very good were grouped to increase the representation of participants in these categories. Thus, the final classification adopted for the IGAF as well as for the individual tests had three levels, namely, very weak/weak, fair, and good/very good.

The tests were applied in the following order: (1) sitting to standing, (2) elbow flexion, both for muscle strength evaluation, (3) time up and go (TUG), for analysis of sitting, balance, position transfers, stability, and change of gait course, and (4) a 6-min walk (for aerobic capacity).

### Stroop test

The adapted version of the Stroop test was used as a tool to assess the executive function of the volunteers. The validated model by Kulaif and Valle (2008) with numbers and colors, adapted from the Victoria version (Tremblay et al., 2016), is very useful in the presence of unlettered participants. Prior to the test's beginning, it was assured that the participant was able to recognize/distinguish the colors and numbers of the test without hesitation, to validate the results. The test consists of 4 cards. In the first (card 1—colors), the participant is asked to quickly name the colors rectangles (red, green, blue, and black). Then, in the second card (card 2—colors and numbers) numbers were added to the colored rectangles like in card 1, at this moment the participant is asked to name the respective number or the color of the rectangles. The third card (card 3—Stroop Effect) consists of colored numbers (same colors as card 1), the participant is then asked to name the colors of the number or the respective number when they were black. At this stage, a certain degree of interference may be unavoidable. Thus, to further minimize any frustration, the last card (card 4) with only numbers colored in black, that should be named was added to our model. The evaluation process considers the time spent on each card, as well as the number of errors. When the error or spontaneous self-corrected, we considered it right.

### ACTN-3 polymorphism analysis

Epithelial cells from oral mucosal were collected by mouth scraping (buccal swab). DNA extraction was performed following the chelex resin protocol for mouth swabs (Ostheim et al., 2020; Yan et al., 2022). Quantification was performed on a NanoDrop^®^ spectrophotometer – ND1000. The DNA used in the standard amplification reactions was diluted in autoclaved ultrapure water. The analyzes of the selected genotypes were performed by the allelic discrimination method in the real-time-PCR (Polymerase Chain Reaction) equipment, using Taqman^®^ SNP genotyping assays (Applied Biosystems, Foster City, CA).

### Statistical analysis

To characterize the sample, education, nutritional status, functional fitness, and respective GFFI, the general linear model (GLM) or Chi-square test was used. For GLM, the results are presented as the mean and standard deviation of the mean followed by the *F* value. The Chi-square test was conditioned on the interpretation of the residual (R = observed value minus the expected value) and the adjusted residual (RA), when significant, followed by the *X*^2^ value. To analyze the effect of genotype on muscle strength and aerobic capacity, ANCOVA analysis was used, with age as a covariant. As for executive function and the effect of functional fitness on the response to the Stroop test, a linear regression was performed with the following independent variables: age, schooling, sex, genotype and the tests: elbow flexion, sitting and standing, and walking for 6 min. For the elbow flexion and sit and stand tests, the number of repetitions was analyzed, while for the 6-min walk, we used the distance in meters. The analysis of the models was performed through the interpretation of the standardized and adjusted coefficient (β), followed by the analysis of the *t* value and significance value. When the independent variables presented more than one possibility of classification, we fixed one of them. For all statistical tests, it was adopted a level of significance lower than 5% (*p* ≤ 0.05).

## Results

Table 1 presents the descriptive data of the sample. The genotype distribution of the ACTN3 R557X polymorphisms was 110 (31%) for the RR genotype, 148 (42%) for the RX genotype, and 89 (25%) for the XX. The mean age between the groups was similar, with no statistically significant difference. Nutritional variables and schooling also showed no significant difference between the genotypes studied.

**Table 1:** Descriptive characteristics of the study.

	**General**	**RR**	**RX**	**XX**	***F(p)***	**χ^2^(p)**
***N***	347	110	148	89		
**Age (years)**	71.0 ± 6.28	70.5 ± 6.07	71.2 ± 6.21	71.7 ± 6.70	0.37 (0.68)	
**IMC (kg/m** ^2^ **)**	27.9 ± 4.63	27.9 ± 4.30	27.4 ± 4.71	28.6 ± 4.83	1.80 (0.16)	
**Nutritional status**						8.45 (0.20)
Underweight (%)	9.5	6.4	14.2	5.6		
Eutrophic (%)	36.0	35.5	35.8	37.1		
Overweight (%)	24.2	29.1	21.6	22.5		
Obese (%)	30.3	29.1	28.4	34.8		
**Educational level (years of study)**						8.86 (0.35)
Never studied (%)	23.3	27.3	22.3	20.2		
1–3 (%)	22.5	26.4	20.3	21.3		
4–8 (%)	11.5	10.1	10.1	15.7		
9–11 (%)	23.9	24.5	23.6	23.6		
>12 years (%)	18.7	11.8	23.6	19.1		

Data expressed by percentage (%) when categorical and by mean and standard deviation (M + DP) when continuous. F, Fisher (referring to GLM test value); X^2^, Chi-square.

The functional fitness of the participants was assessed using the Senior Fit test. In our analysis, we used the elbow flexion, sit and stand, TUG, and 6-min walk tests. To increase the representation of participants in the categories, some classifications were grouped. Thus, the classification adopted in this study had three categories: very weak/weak, regular, and good/very good. In Table 2, we see the results of functional fitness. There was no significant difference between the groups. It is possible to see that the majority of the elderly were classified as regular in all genotypes. Indicating that there is no interference of the genotype in the classification of functional fitness. When verifying the number of repetitions in the lower and upper limb strength tests and the walked distance (meters) in the aerobic test, we also did not observe a significant effect of the genotype (Table 3). The prevalence of falls in the elderly is also an indication of the individual's functional quality. Thus, we collected information on 12-month fall recall, but there was no genotype effect on this variable.

**Table 2:** Effect of genotype on functional fitness classification.

	**General**	**RR**	**RX**	**XX**	**χ^2^(*p*)**
***N***	347	110	148	89	
**GFFI**					3.37 (0.49)
Very weak/weak	31.70	2.70	4.70	1.10	
Regular	42.70	65.50	58.80	65.20	
Good/very good	25.60	31.80	36.50	33.70	
**Elbow flexion**
Very weak/weak	7.5	7.3	8.1	6.7	0.52 (0.97)
Regular	7.2	6.4	8.1	6.7	
Good/very good	85.3	86.4	83.8	86.5	
**Sit-to-Stand**					3.21 (0.52)
Very weak/weak	34.0	29.1	35.1	38.2	
Regular	25.4	30.0	22.3	24.7	
Good/very good	40.6	40.9	42.6	37.1	
**TUG**					6.43 (0.17)
Very weak/weak	64.0	66.4	63.5	61.8	
Regular	21.6	24.5	17.6	24.7	
Good/very good	14.4	9.1	18.9	13.5	
**6-min walk**
Very weak/weak	57.3	55.5	57.4	59.6	8.60 (0.07)
Regular	22.8	28.2	16.9	25.8	
Good/very good	19.9	16.4	25.7	14.6	
**12-months fall recall**					4.49 (0.10)
Yes	12.7	10.9	10.1	19.1	
No	87.3	89.1	89.9	80.9	

Data expressed by percentage (%). X^2^, Chi-square; GFFI, General functional fitness index.

**Table 3:** Effect of genotype on strength and aerobic endurance.

	**RR**	**RX**	**XX**	**F (*p*)**
Elbow flexion	23.1 ± 5.80	23.4 ± 6.34	23.5 ± 7.09	0.25 (0.77)
Sit-to-Stand	16.0 ± 3.22	15.8 ± 6.68	15.4 ± 3.41	0.59 (0.55)
6-min walk	516 ± 99.7	535 ± 106	518 ± 87.5	1.94 (0.14)

Executive function was assessed through the Stroop test. We performed a linear regression to verify the factors that influence the volunteers' executive functions (Table 4). We can observe that age and education are factors that influence the answer's time in card 3. As the person ages, the response time for card 3 increases (*p* = 0.003). Regarding education, the higher the educational level, the better the response in the Stroop test (*p* < 0.001). As for the genotype, RR individuals have a worse cognitive performance (they answer the test for a longer time) when compared with RX and XX individuals (*p* = 0.05 for both) (Tables 4, 5). This result suggests that the presence of the X allele of the ACTN3 gene is related to a better performance of the executive function.

**Table 4:** Predictor factors for executive function in the elderly.

	**β**	***t***	***p***	**CI 95%**
Age	0.69	4.01	**<0.001**	−17.48 to 35.08
**Educational level**
2–1	−6.66	−2.27	**0.02**	−12.42 to −0.89
3–1	−5.98	−1.62	0.16	−13.24 to 1.27
4–1	−15.31	−5.09	**<0.001**	−21.23 to −9.40
5–1	−17.58	−5.24	**<0.001**	−10.99 to −5.24
Sex	−3.71	−1.70	**0.08**	−8.00 to 0.57
**Genotype**
RX—RR	−4.55	−1.92	**0.05**	−9.17 to 0.05
XX—RR	−5.17	0.02	**0.05**	−10.38 to 0.02

Adjusted Linear Regression Model (β) - Stepwise method, Dependent variable: time on Card 3, Independent variables—sex, education, genotype.

Bold values mean that they were statistically significant.

**Table 5:** Stroop test performance.

	**RR**	**RX**	**XX**	
***n***	110	148	89	F (*p*)
**Card 1**
Time (seconds)	20.6 ± 11.8	19.2 ± 6.99	19.2 ± 6.46	
Errors (unity)	0.30 ± 1.06	0.22 ± 0.82	0.05 ± 0.27	
**Card 2**
Time (seconds)	22.5 ± 12.3	22 ± 9.68	20.5 ± 7.34	
Errors (unity)	0.40 ± 0.99	0.45 ± 1.25	0.30 ± 0.85	
**Card 3**
Time (seconds)	47.1 ± 25.6	41.6 ± 15.9	41.4 ± 19.7	3.50 (0.03)
Errors (unity)	3.39 ± 3.88	2.82 ± 3.49	2.81 ± 3.52	
**Card 4**
Time (seconds)	15.4 ± 10.1	13.9 ± 5.76	13.6 ± 4.61	
Errors (unity)	0.09 ± 0.53	0.05 ± 0.30	0.11 ± 0.76	

Data expressed as mean and standard deviation (M ± SD).

## Discussion

The present study aimed to investigate the effect of the R577X polymorphism of the ACTN-3 gene on functional fitness and executive function in the elderly. For this, we recruited 347 volunteers, both sexes, aged 60 years old and over. The R577X polymorphism results in the change of the R allele for a premature stop codon X, which leads to protein deficiency, thus influencing muscle phenotype (Pickering and Kiely, 2018). In our studied population, we did not observe any differences between functional fitness tests and the different genotypes of the ACTN-3 gene. Even though the presence of the X allele is associated with decreased physical performance, increased frailty (Ma et al., 2018), loss of muscle function (Kikuchi et al., 2015) and an increased risk of sarcopenia (Cho et al., 2017), our volunteers showed similar performance regardless of individual genotype—both in muscle strength tests (elbow flexion, sit and stand) and in aerobic capacity tests (6-min walk). Similarly, a cohort study that evaluated the association between the ACTN-3 gene and different muscle phenotypes in more than a thousand volunteers found no influence of the ACTN-3 genotype on muscle phenotypes (Delmonico et al., 2008). However, when evaluated over 5 years, a decline in muscle function with a significant genotypic association could be seen. Individuals with the XX genotype showed a greater decline in muscle function (Delmonico et al., 2008).

As the volunteers in our sample belong to an elderly community center where they participate in various sports activities, regular training may have conferred benefits on the physical integrity of the elderly, regardless of their genetic predispositions. As found in the study by Moraes et al. (2018), differences in muscle strength were not found between the genotypes of the ACTN-3 gene, however, after a physical exercise intervention, there was an improvement in muscle function even in individuals with protein deficiency (Moraes et al., 2018). These findings indicate that the regular practice of physical activity in the elderly population is capable of attenuating possible muscle losses related to the ACTN-3 gene polymorphism. Reinforcing the importance of good maintenance of muscle function for the elderly.

In addition, muscle strength has been studied as a predictor of cognitive performance in aging. In a recent study carried out by our group, we observed that functional fitness, in addition to age and education, was one of the predictors of the cognitive performance of the elderly, assessed by the Stroop test (Braga et al., 2022). Among fitness tests, muscle strength appears as a potential predictor of a better cognitive response (Braga et al., 2022). The mechanism by which muscle strength influences cognition is still not well-understood. However, several studies show a relationship between these two variables. In a Brazilian study, 62 participants underwent 24 weeks of strength exercise and, after the intervention, tests of executive function as well as short- and long-term memory were applied. It was observed that volunteers who participated in the intervention showed improvement in neuropsychological tests (Cassilhas et al., 2007). The same association was found in the Koreans elderlies. This longitudinal study, which followed participants for 6 years, suggests that poor handgrip strength is significantly associated with an increased risk of cognitive dysfunction in aging (Jeong and Kim, 2018). However, none of these studies evaluated a genetic component along with physical capabilities in the cognitive response during aging.

Based on this, we sought to investigate whether the ACTN-3 gene can be a predictive factor for the executive response in the elderly. As individuals with the RR genotype have characteristics of greater muscle strength, we hypothesized these individuals would perform better in executive function. Interestingly, participants with the RR genotype did not have a good executive performance, answering the test in a longer time when compared to individuals who had the ACTN-3 (XX) gene polymorphism. As one of the characteristics of the XX genotype is a better cardiorespiratory performance, it may indicate that these individuals are more likely to present positive cognitive results, since a good aerobic capacity contributes to healthy brain aging (Hayes et al., 2013). The prevalence of the XX genotype is increased in the population as a result of the evolutionary adaptations to cold climate regions (Wyckelsma et al., 2021). Therefore, this could be a speculative suggestion that the effect on cognitive performance could have been derived from a positive selection of the X allele. Also, as the glycolytic fibers present accentuated degradation with aging (Milikovic et al., 2015), the effects of the RR genotype on cognition in older individuals may not be so apparent. These results are interesting and shed light on further investigation, for instance, it would be interesting to investigate these relationships in different domains of cognition. Due to the number of participants and research volunteers that worked in our study, we were unable to perform further cognitive analyses. For this reason, we limited our evaluation to the executive function. Also, it would be interesting to investigate other genetic factors that may be involved in muscle characteristics. The ACTN-2 gene, for example, in some cases, can partially supply the deficiency of the α-actinin-3 protein (MacArthur et al., 2007), and in this case, may be responsible for maintaining physical function. The knowledge of genetic markers or factors that can influence the cognition and functional fitness of the elderly, can be helpful for the creation of personalized intervention strategies to suit the individual needs of this population.

## Data availability statement

The original contributions presented in the study are included in the article/supplementary material, further inquiries can be directed to the corresponding author/s.

## Ethics statement

The studies involving human participants were reviewed and approved by Comitê de Ética em Pesquisa – UNIFESP (CEP/UNIFESP – n:0630/2018). The patients/participants provided their written informed consent to participate in this study.

## Author contributions

JS participated in the study design, data collection and analysis, interpretation of results, discussion, drafting, and manuscript writing. PG, SS, and RA participated in the study design, data collection, and intellectual contribution. NS and ID participated in the data analysis. CM participated in data analysis, interpretation of the results, and critical review. SG participated in the supervision of the study, data interpretation, and critical review. All authors contributed to the article and approved the submitted version.

## Funding

This study was supported by Coordenação de Aperfeiçoamento de Pessoal de Nível Superior (CAPES), Conselho Nacional de Desenvolvimento Científico e Tecnológico (CNPq), Fundação de Amparo ao Ensino e Pesquisa (FAEP), and Fundação de Amparo à Pesquisa do Estado de São Paulo (FAPESP #2014/00035-1; #2018/16565-0), FAPESP #2017/04528-0; #2017/26075-8, CAPES-PRINT (#88881.310490/2018-01, and Conselho Nacional de Desenvolvimento Científico e Tecnológico (CNPq #301732/2018-3; CNPq #408676/2018-3).

## Conflict of interest

The authors declare that the research was conducted in the absence of any commercial or financial relationships that could be construed as a potential conflict of interest.

## Publisher's note

All claims expressed in this article are solely those of the authors and do not necessarily represent those of their affiliated organizations, or those of the publisher, the editors and the reviewers. Any product that may be evaluated in this article, or claim that may be made by its manufacturer, is not guaranteed or endorsed by the publisher.
